# Identification of a Regulatory T Cell Specific Cell Surface Molecule that Mediates Suppressive Signals and Induces Foxp3 Expression

**DOI:** 10.1371/journal.pone.0002705

**Published:** 2008-07-16

**Authors:** Rui Wang, Qi Wan, Lina Kozhaya, Hodaka Fujii, Derya Unutmaz

**Affiliations:** 1 Department of Microbiology, New York University School of Medicine, New York, New York, United States of America; 2 Department of Pathology, New York University School of Medicine, New York, New York, United States of America; Instituto Oswaldo Cruz and FIOCRUZ, Brazil

## Abstract

Regulatory T (T_reg_) cells control immune activation and maintain tolerance. How T_regs_ mediate their suppressive function is unclear. Here we identified a cell surface molecule, called GARP, (or LRRC32), which within T cells is specifically expressed in T_regs_ activated through the T cell receptor (TCR). Ectopic expression of GARP in human naïve T (T_N_) cells inhibited their proliferation and cytokine secretion upon TCR activation. Remarkably, GARP over-expression in T_N_ cells induced expression of T_reg_ master transcription factor Foxp3 and endowed them with a partial suppressive function. The extracellular but not the cytoplasmic region of GARP, was necessary for these functions. Silencing Foxp3 in human T_reg_ cells reduced expression of GARP and attenuated their suppressive function. However, GARP function was not affected when Foxp3 was downregulated in GARP-overexpressing cells, while silencing GARP in Foxp3-overexpressing cells reduced their suppressive activity. These findings reveal a novel cell surface molecule-mediated regulatory mechanism, with implications for modulating aberrant immune responses.

## Introduction

Regulatory T cells (T_regs_) were recently identified as a specialized subset of T cells that suppress auto-reactive cells to maintain immunological tolerance and inhibit autoimmunity [1]. T_regs_ have also been shown to control excessive or chronic immune activation after infections with various pathogens [2]. Naturally occurring T_regs_ are thought to be generated in the thymus, express high levels of CD25 and the transcription factor forkhead box protein P3 (Foxp3), and constitute about 5–10% of periphery total CD4^+^ T cells in mice [1]. Human and murine T_regs_ share similar characteristics, such as hypo-responsiveness to T cell receptor (TCR) activation and mediation of their suppressive activity through cell-contact-dependent mechanisms [1]. Foxp3 is critical in T_reg_ development and function. Mice that are deficient in Foxp3 develop severe autoimmune diseases, and mutations of human Foxp3 gene result in immunodysregulation, polyendocrinopathy, enteropathy, X linked syndrome (IPEX), which also is characterized by severe autoimmunity [1], [2].

Although the characteristics and function of T_regs_ have been extensively studied, mechanisms of how T_regs_ mediate their suppressive activity are still unclear. Various mechanisms have been proposed, such as cytokine deprivation-induced apoptosis [3], secretion of inhibitory cytokines [4], [5], manipulation of antigen presenting cells (APCs)[6], and recruitment of mast cells [7]. Several molecules have also been proposed to be important in T_reg_ generation, development or function, such as glucocorticoid-induced TNF receptor (GITR)[8], indoleamine 2,3-dioxygenase (IDO)[9], Heme oxygenase-1 (HO-1)[10], lymphocyte activation gene-3 (LAG-3)[11]. How these diverse sets of molecules regulate T_reg_ cell differentiation and suppressive function remain unresolved.

In this report, we identified a T_reg_ cell specific cell surface molecule called GARP or LRRC32. Ectopic expression of GARP in primary human T cells remarkably induced Foxp3 expression and endowed them with partial T_reg_ phenotype, including impairment of proliferative capacity and acquisition of a suppressive activity. Structure-function analysis of GARP revealed that the extracellular domain, but not the cytoplasmic region, was important for its function, suggesting an interaction of GARP with other signaling receptor(s). Thus, determining the role of this novel molecule in T_reg_ mediated suppression could be important in understanding tolerance mechanisms and exploiting its function for regulating immune responses.

## Results and Discussion

### Identification of a cell surface molecule expressed on activated T_reg_ cells

**Figure 1:**
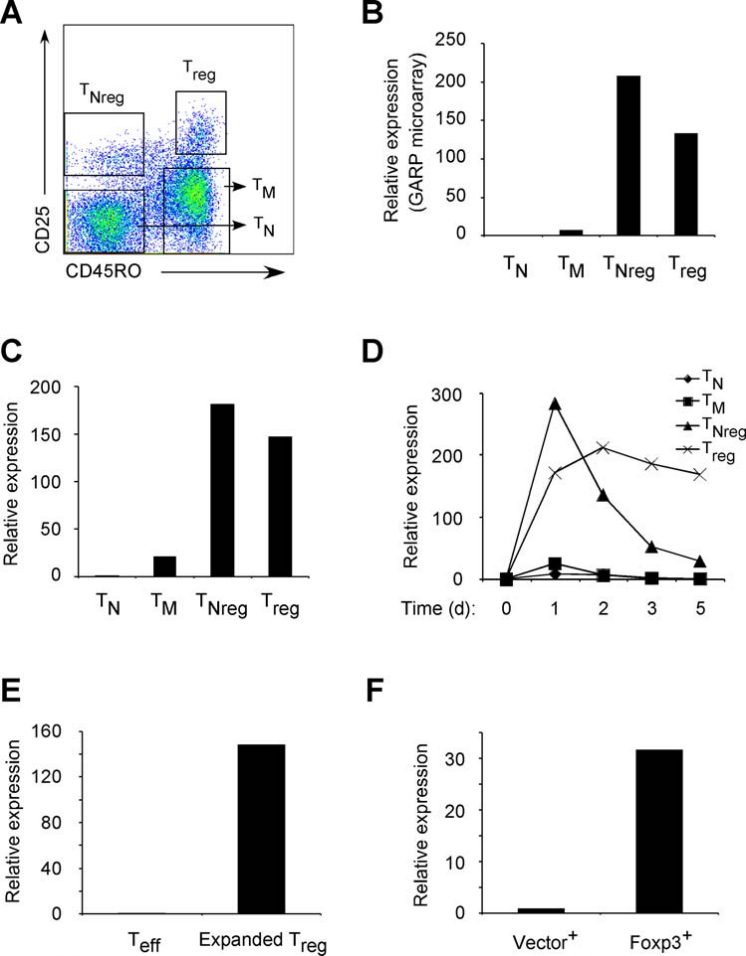
GARP is specifically expressed in activated T_reg_ cells. (A) Representative T cell subset staining from normal donor. CD4^+^ T cells isolated from human PBMC were stained with anti-CD25 and CD45RO antibodies. Naïve (T_N_), memory (T_M_), T_Nreg_ and T_reg_ cells were identified as shown in brackets. (B) GARP levels of recently activated T cell subsets from the microarray. (C) GARP levels of different T cell subsets 1 day post TCR activation as determined by qPCR. One representative experiment of five donors is shown. (D) Kinetics of GARP levels in different T cell subsets at different time points before and after TCR stimulation. Diamond, T_N_; square, T_M_; triangle, T_Nreg_; cross, T_reg_. d, day. One experiment representative of three donors is shown. (E) Expanded T_reg_ cells from T_Nreg_ precursors and (F) Foxp3-transduced cells upregulate GARP after TCR stimulation. Cells were *in vitro* expanded for two weeks before subjecting to qPCR analysis. T_eff_, expanded T_N_ cells; Vector^+^, control vector-transduced T_N_ cells; Foxp3^+^, Foxp3-transduced T_N_ cells. One experiment representative of three donors is shown.

In order to identify novel molecules that could be involved in human T_reg_ development and function, we first isolated human T cell subsets as previously described [12]. The purified CD4^+^ T cells from healthy donors, were divided into four subsets based on CD45RO and CD25 staining: 1) T_regs_ (CD45RO^+^CD25^hi^) 2) Memory T (T_M_) cells (CD45RO^+^CD25^−^) 3) Naïve T (T_N_) cells (CD45RO^−^CD25^−^) and 4) Naïve subset of T_reg_ (T_Nreg_) cells (CD45RO^−^CD25^hi^)[12]–[15] as shown in Fig. 1A. The fourth group of human T cell subset, T_Nreg_ cells, which was recently characterized, displays an intermediate suppressive capacity *ex vivo* but can be expanded *in vitro* to develop into fully mature T_regs_ [12]–[15]. All four groups of purified T cell subsets were then stimulated through the T cell receptor (TCR) overnight and RNA was extracted for DNA gene chip analysis to identify T_reg_ specific genes. The DNA microarray analysis led to identification of a cell surface molecule that was expressed more than 100 fold in both T_Nreg_ and T_regs_ as compared to T_N_ or T_M_ cells (Fig. 1B). This 662 amino acid (aa) encoding gene, originally called *GARP* or *LRRC32*, was found to be expressed at high levels in placenta, lung and kidney [16], [17].

To confirm the high expression level of GARP in activated T_regs_, we performed quantitative real time RT-PCR (qPCR) analysis of GARP gene expression in all T cell subsets stimulated through the TCR. Consistent with the microarray data, qPCR also showed that the expression levels of GARP in both T_Nregs_ and T_regs_ were more than 100 fold higher as compared to T_N_ cells and about 10 fold higher than T_M_ cells (Fig. 1C).

To determine the kinetics of GARP expression in different T cell subsets, qPCR analysis for GARP expression was performed in T_N_, T_M_, T_Nreg_ or T_reg_ cells either at a resting state or at different time points post-activation. We found that GARP is rapidly upregulated after TCR stimulation in both T_Nregs_ and T_regs_ (Fig. 1D). Interestingly, high level of GARP persisted for at least 5 days on T_regs_, but declined in T_Nregs_ within 2 days. This could be because T_Nregs_ are not fully mature and have to undergo a differentiation program to sustain GARP expression [12]. However, post *in vitro* expansion, T_Nreg_ cells acquire complete T_reg_ characteristics both phenotypically and functionally [12]. Indeed, *in vitro* expanded T_Nregs_ (hereafter referred as T_regs_) also upregulated GARP expression post TCR activation and exhibited kinetics of GARP expression that were similar to T_regs_ directly isolated from peripheral blood (Fig. 1E and data not shown). Furthermore, ectopic expression of Foxp3 in T_N_ cells, which can program them into T_regs_ [18] also upregulated GARP mRNA expression (Fig. 1F and data not shown). Taken together these findings suggest that GARP expression is specifically induced on natural or reprogrammed T_regs_ upon TCR stimulation, and might be involved in maintenance of T_reg_ characteristics or T_reg_-mediated suppressive activity.

### Overexpression of GARP endows T_N_ cells with partial T_reg_ phenotype and function

**Figure 2:**
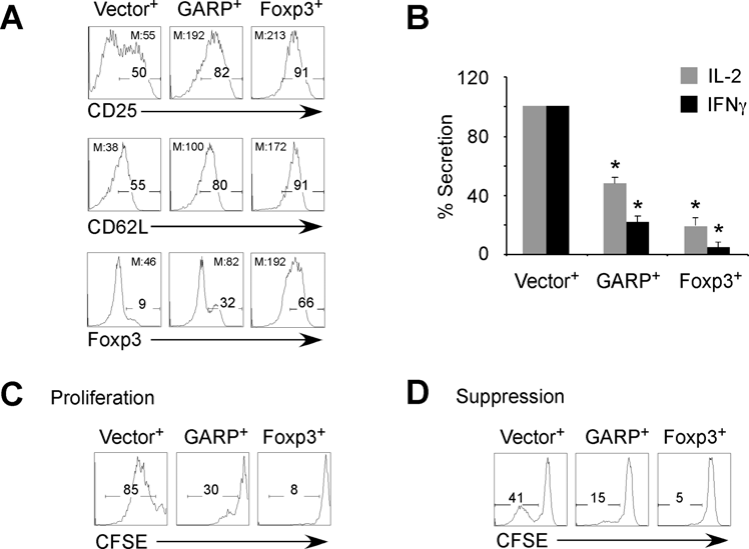
Ectopic expression of GARP endows T_N_ cells with partial T_reg_ phenotype and function. (A) GARP-transduced cells express higher levels of CD25 and CD62L, and upregulate Foxp3 at a greater level after TCR stimulation than the control vector-transduced cells. Cells were transduced and expanded for two weeks before staining for CD25 and CD62L. RFP^+^ cells were then sorted and re-stimulated by DC plus SEB at suboptimal conditions before staining for Foxp3. Vector^+^, control vector-transduced T_N_ cells; GARP^+^, GARP-transduced T_N_ cells; Foxp3^+^, Foxp3-transduced T_N_ cells. M, Geometric mean. Data are representative experiments from at least three different donors. (B) GARP-transduced cells produce reduced levels of cytokines. Cytokine production in the supernatant of the activation culture was measured using CBA and data are shown by first normalizing the control geometric mean to be 100%. Range of control production (pg/ml): IL-2, 300–1000; IFNγ, 3000–10000. Data represent the mean±SEM of experiments from at least three different donors. *, *P*<0.05, n≥3; experimental versus control. (C) GARP-transduced cells exhibited impaired proliferative capacity and (D) intermediate suppressive activity. Data are representative experiments from at least three different healthy human donors.

To explore the role of GARP in T_reg_ development and function, we subcloned human GARP into a HIV-derived vector (HDV) that encodes red fluorescent protein (RFP) as a marker [18]. T_N_ cells were isolated as described above, activated by monocyte-derived dendritic cells (DC) plus superantigen (SEB), and transduced with HDV encoding GARP, Foxp3 or vector only as control. Expression of GARP and Foxp3 in transduced cells was confirmed by qPCR and intracellular staining respectively (data not shown). The transduced cells were expanded in IL-2 containing media for two weeks, and stained for CD25 and CD62L, which are known to be expressed at higher levels on naturally occurring T_regs_ or on Foxp3-overexpressing T cells [19], [20]. GARP-transduced cells expressed higher levels of CD25 and CD62L than the control vector-transduced cells, but the expression was lower compared to the Foxp3-transduced cells (Fig. 2A). Remarkably, GARP-transduced cells expressed higher level of Foxp3 compared to the cells transduced with the control vector after re-activation with DCs pulsed with SEB (Fig. 2A).

To determine whether GARP-expressing T cells display functional properties of T_regs_, transduced cells were sorted on the basis of RFP expression and stimulated through DCs pulsed with SEB at suboptimal concentrations. Cytokine production in the activation culture was assessed. T cells ectopically expressing GARP secreted lower levels of IL-2 and IFNγ compared to control vector-transduced T cells (Fig. 2B). To assess the proliferative capacity of GARP-expressing cells, transduced cells were labeled with Carboxyfluorescein succinimidyl ester (CFSE) and stimulated through TCR. GARP-expressing cells also displayed decreased proliferative capacity as compared to the T cells expressing vector only, when stimulated with DC and suboptimal SEB (Fig. 2C). Furthermore, GARP-expressing cells displayed some suppressive activity against T_N_ targets, although this was not as potent as Foxp3-transduced cells (Fig. 2D). Taken together, our data indicate that T cells ectopically expressing GARP acquire a partial phenotypic and functional profile of T_regs_.

### GARP function requires the extracellular region but not the cytoplasmic portion

**Figure 3:**
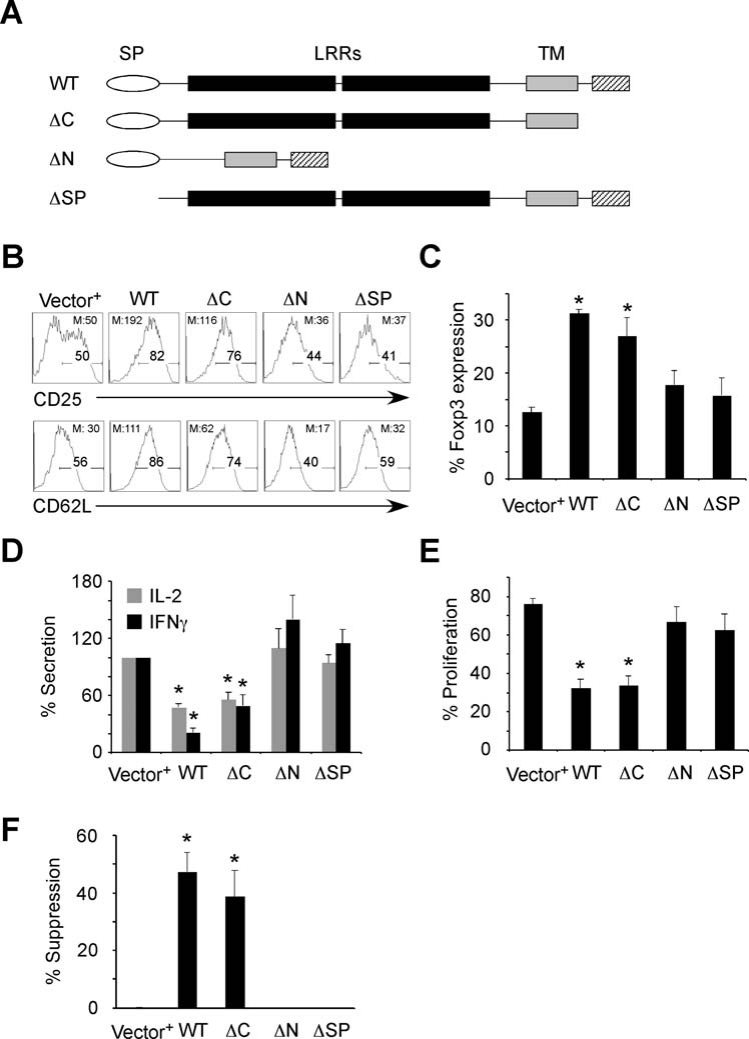
Phenotypic and functional analysis of T cells ectopically expressing GARP mutants. (A) Schematic of WT and mutant GARPs. SP, signal peptide; LRR, leucine-rich repeat; TM, transmembrane domain. (B) Both ΔN and ΔSP mutant-transduced cells do not sustain high levels of CD25 and CD62L expression and (C) Foxp3 expression post-activation as determined by staining. Cells were transduced and prepared as described in Fig. 2. M, Geometric mean. (D) Cytokine profiles of WT and mutant GARP-transduced cells. Cytokine production in the supernatant of the activation culture was measured using CBA kit (BD Biosciences) and percent secretion in Y-axis is calculated by first normalizing the secretion of cytokines from control cells to 100%, and calculating the amount of cytokine secreted from other cell conditions relative to this control. This representation allowed us to combine data from multiple donors in the same figure. This normalization Data represent the mean±SEM of experiments from at least three different donors. *, *P*<0.05; experimental versus control. Range of production (pg/ml): IL-2, 0–1000; IFNγ, 0–10000. (E) Proliferative capacity is shown as percentage of proliferated cells as determined by dilution of CFSE 4 or 5 days post re-stimulation. (F) Percentage of suppression was calculated based on CFSE dilution of target T_N_ cells in the suppression culture. The % suppression on Y-axis is calculated as follows: We first determined the percent of CFSE labeled target cells dividing in response to the stimuli alone. We then determined the proliferative percentage of cells in the presence of suppressor (T_regs_ or GARP expressing cells) or control effector T cells. The percent suppression was then calculated by percent reduction in proliferation of the target cells in the presence of effectors as compared to target cells alone. As an example, in one experiment 40% of the CFSE labeled T cells divided in response to SEB stimulation. In the presence of T_regs_ the same cells divided only 4%. Thus, we represent this as 90% suppression. Data represent the mean±SEM of experiments from at least three different donors. * *P*<0.05; experimental versus control.

The *GARP* gene encodes a cell surface protein with a signal peptide (SP) for cell surface translocation [21], an extracellular region that is composed of 20 leucine-rich repeats (LRRs), a transmembrane domain (TM) and a short cytoplasmic region of 15 aa [17], [22]. LRR domains in other proteins are involved in a variety of functions, principally through protein-protein interaction [23]. Because GARP has a very short cytoplasmic region, we hypothesized that only the extracellular LRRs and the SP are required for its function. Accordingly, we prepared three different mutants: 1) Deletion of the cytoplasmic tail (ΔC), 2) Deletion of all LRRs in the N-terminus (ΔN), and 3) Deletion of the SP at the N-terminus (ΔSP). These mutants are shown schematically in Fig. 3A. All mutants were sequence confirmed and subcloned into the HDV, and lentiviral vectors were generated to transduce primary T_N_ cells. Phenotypic analysis revealed that T_N_ cells expressing the ΔC mutant showed similar upregulation of CD25, CD62L and Foxp3 expression compared to the WT GARP, whereas T cells expressing the ΔN or ΔSP GARP mutants had lost this activity (Fig. 3B and 3C).

Next, we compared proliferative capacity, cytokine production and suppressive activity of the mutants to the WT GARP. Similar to the surface phenotype, T_N_ cells transduced with the ΔC mutant exhibited impaired proliferative capacity, reduced cytokine production, and enhanced suppressive capacity comparable to WT GARP-expressing cells (Fig. 3D–F). However, the ΔN and ΔSP mutant-expressing cells did not display any of the inhibitory functions of GARP-expressing cells (Fig. 3D–F). Together, these data indicate that both the SP and the LRRs in the extracellular portion of GARP, but not the cytoplasmic domain, are crucial to its function.

### Does GARP mediate its inhibitory function through expression of Foxp3?

**Figure 4:**
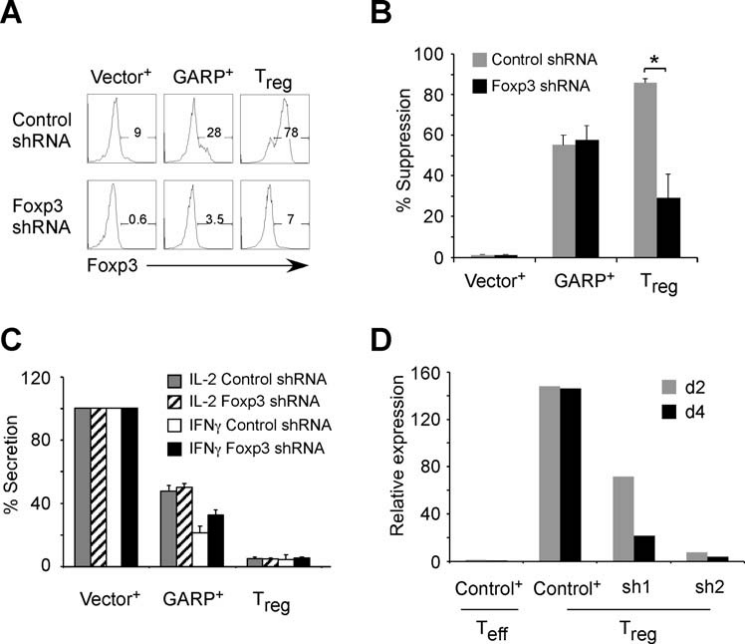
GARP functions as a downstream effector molecule of Foxp3 expression. (A) Efficient knockdown of Foxp3 expression in GARP-transduced and T_reg_ cells. T_N_ cells were co-infected with shRNA-expressing HDV (GFP) and control or GARP-expressing HDV (RFP), while T_regs_ from the same donor were transduced with shRNA-expressing HDV. Cells were cultured for two weeks before staining for CD25 and CD62L, and before sorting for experimental lines: GFP^+^RFP^+^ for control vector or GARP-expressing cells; GFP^+^ for T_regs_. Foxp3 was stained after re-stimulation by DC and SEB. Vector^+^, control vector-transduced T_N_ cells; GARP^+^, GARP-transduced T_N_ cells; T_eff_, expanded T_N_ cells. (B) Suppressive activity and (C) cytokine profiles of Foxp3 shRNA-expressing GARP-transduced and *in vitro* expanded T_reg_ cells. Data represent the mean±SEM of experiments from three different donors. Cytokine data are shown by first normalizing the control to be 100%. Range of control production (pg/ml): IL-2, 300–1000; IFNγ, 3000–10000. *, *P*<0.05, n≥3; experimental versus control. (D) GARP level in *in vitro* expanded T_reg_ cells was decreased by silencing Foxp3 as determined by qPCR analysis day 2 or day 4 post TCR stimulation. d, day. Two different shRNA oligos against Foxp3 were tested, both of which have been described before [12].

We have shown that GARP was upregulated in freshly isolated T_Nreg_, T_reg_ and Foxp3-expressing cells as well as *in vitro* expanded T_regs_ (Fig. 1). These findings suggest that GARP is a potential downstream effector molecule in T_regs_. However, T cells ectopically expressing GARP also upregulate Foxp3 expression in a sizeable portion of these cells (Fig. 2A). Therefore, it is possible that functional and phenotypic changes observed upon GARP overexpression could be indirectly due to induction of Foxp3, which then induces other effector molecules. To address this possibility we performed an experiment to silence Foxp3 expression in GARP-transduced cells using shRNA against Foxp3. Cells were then expanded for two weeks and efficient knockdown of Foxp3 expression was confirmed (Fig. 4A). As shown in Fig. 4B, silencing Foxp3 in GARP-expressing cells did not alter their suppressive ability, while downregulation of Foxp3 in T_regs_ resulted in significant attenuation of their suppressive capability [12], which was reduced to similar levels as GARP-expressing cells (Fig. 4B). Moreover, GARP-expressing cells produced reduced levels of cytokines even in the presence of Foxp3 shRNA (Fig. 4C), which was similar to control shRNA expressing cells (data not shown). In addition, silencing Foxp3 expression in T_reg_ cells reduced induction of GARP upon TCR activation (Fig. 4D), which correlated with partial loss of suppressive activity in these cells (Fig. 4B). These findings suggest that induction of GARP in T_regs_ is dependent on Foxp3 expression, while its inhibitory action on T cell activation is independent of sustained expression of Foxp3.

### Silencing GARP in Foxp3-induced T_reg_ cells attenuate their suppressive activity

**Figure 5:**
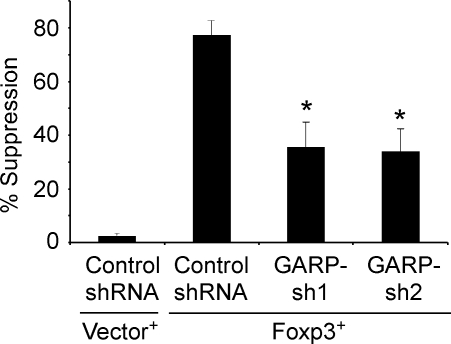
Silencing GARP in Foxp3-induced T_regs_ attenuate their suppressive activity. CD4+ T cells were co-transduced with shRNA-expressing or control lentiviruses (GFP+) and Foxp3-expressing lentiviral vectors (RFP+). GFP+ RFP+ T cells expressing both shRNA for GARP and Foxp3, were first sorted to high purity through FACS. The suppressive activity of these cells were then determined using CFSE assay and represented as percent suppression as described in detail in Figure 3. Data are representative experiments from two different donors and shows independent results from two separate shRNAs against GARP. Data represent the mean±SEM.

Induction of GARP expression requires the expression of Foxp3 while it can mediate its function independent of Foxp3 expression. Based on these findings we hypothesize that GARP is an effector molecule of suppression downstream of Foxp3 expression. To test this hypothesis further, we silenced GARP expression through shRNA in Foxp3-induced Tr_egs_. We designed two different shRNAs against GARP, which reduced the expression of GARP between 60 to 80% in Foxp3-induced T_regs_ (data not shown). We found that expression of GARP-shRNA in Foxp3-induced T_regs_ attenuated their suppressive activity as compared to the control shRNA expressing cells (Fig. 5). This important result supports our hypothesis that a sizeable part of suppressive activity mediated by Foxp3 is through the induction of GARP in T_reg_ cells.

## Discussion

Naturally occurring T_regs_ are crucial to the establishment of immunological tolerance. Despite intense research, the mechanisms by which T_regs_ mediate their suppressive effect are not completely understood. Here we identified a molecule, called GARP, which mediates part of the T_reg_ suppressive activity. Ectopic expression of GARP in human T_N_ cells resulted in a partial T_reg_ phenotype, as determined by increased Foxp3, CD25 and CD62L expression, decreased cytokine production (IFNγ and IL-2), impaired proliferative capacity and display of suppressive activity. Silencing GARP in Foxp3-induced T_regs_ reduced their suppressive function. These data suggest a pivotal role for GARP in controlling aberrant or unwanted T cell activation.

GARP is highly conserved across species, suggesting that it mediates an important function [24]. The mouse and human GARP amino acid sequences share 82% identity. The similarity rises to 87% if conservative amino acid changes are allowed, which argues the possibility for cross-reaction between mouse GARP with human GARP binding partners. Indeed, similar to the human counterpart, murine GARP was also upregulated only in mature murine T_regs_ upon activation (Figure S1). Further, murine GARP-expressing human T cells exhibited a very similar phenotype as those expressing human GARP (Figure S1 C–F). We conclude that both murine and human GARP are able to interact with the human GARP binding partners. Future genetic manipulation in mice to determine the role of GARP *in vivo* will be invaluable in understanding the role of this molecule in function and development of T_reg_ cells.

*GARP* gene, originally called *GARP*, contains two exons. The first exon encodes the signal peptide and nine amino acid residues, and the second one contains 20 repeats of the LRR motif, the transmembrane domain and a short cytoplasmic domain [17], [24]. We found that deletion of LRRs in GARP results in complete loss of its function, while mutant lacking the short cytoplasmic domain behaved similarly to the WT GARP. The LRRs are generally 20–29 residues long, with the consensus sequence LxxLxLxxN/CxL. Proteins with LRR constitute a large superfamily involved in diverse functions, such as adenylate cyclase in yeast, the proteoglycans decorin and biglycan in human, and disease resistance genes in plants [23]. GARP, as an LRR containing molecule (LRRC32), shows similarity to glycoprotein V, which is one of the components forming the GPIb-IX-V complex on platelet surface famous for the interaction with von Willebrand factor complex to mediate the adhesion of platelets to injured vascular surfaces [25]. It was recently shown that GARP is expressed on the cell surface of platelets using a polyclonal antibody raised against its extracellular portion [22]. In addition, we generated GFP-tagged versions of WT and ΔSP mutant of GARP and based on image analysis of cells expressing a GFP, confirmed WT but not ΔSP, appears to be localized to cell surface region (Figure S2). This expression profile of GARP suggests that LRRs at the extracellular region could mediate its function, possibly through interaction with another cell surface molecule(s). We also ruled out that GARP-mediated suppression is mediated by a soluble factor since neither separating GARP-expressing cells via transwells nor adding supernatants from these cells to target cells showed any suppressive function (data not shown). Our finding that the cytoplasmic region was dispensable also strongly argues the possibility that GARP itself is not likely to be delivering intracellular signals but conceivably may interact with a receptor molecule expressed by target T cells, which deliver inhibitory signals. Alternatively, GARP may be part of a signaling surface complex similar to glycoprotein V [25].

We found that GARP was induced in T cells ectopically expressing Foxp3 and reduced in expression when Foxp3 was silenced in natural T_regs_. These findings suggest that Foxp3 is a regulator of GARP expression. It remains to be determined whether Foxp3 directly activates GARP transcription or indirectly through other transcription factors. It will also be interesting to determine whether in IPEX patients or scurfy mice, which have defective expression or mutations in Foxp3, GARP expression is also reduced. Although Foxp3 appears to be required for GARP expression in T cells, silencing Foxp3 in cells forced to express GARP does not reduce their suppressive function. Furthermore, we showed that silencing of GARP in Foxp3-induced T_regs_ diminished their suppressive function. Together these data imply that GARP is an effector molecule downstream of Foxp3 that can directly display inhibitory signaling once it is expressed.

Peripheral tolerance to self-antigens is an important secondary mechanism that prevents autoimmune reactions [26]. In addition, T_regs_ have also been proposed to play a role in pregnancy by allowing continuous release of placental antigens into the maternal circulation, thus permitting tolerance towards the semiallogeneic fetus until birth [9], [27]. Previous studies postulated cellular pathways affected by T_reg_ action including production of inhibitory cytokines, enhanced IDO expression and HO-1 induction [9]. Interestingly, GARP was found to be highly expressed in placenta in both mouse and human [17], [24]. Expression of GARP in placenta might be essential in creating a “tolerant” privileged microenvironment to avoid rejection of the fetus, and this privilege could even be independent of T_regs_, although it remains to be determined whether GARP expression on non-T cells can also inhibit T cell activation.

In summary, GARP is highly specific to activated T_reg_ cells, and has the ability to mediate partial T_reg_ characteristics, importantly including their suppressive function. Identification of this T_reg_ cell specific molecule may have implications in understanding regulation of unwanted immune responses and sustaining tolerance by T_reg_ cells. The discovery of GARP as an immune suppressive molecule in primary human T cells may also be relevant for exploiting the function of this molecule to modulate immune responses during autoimmunity, transplantation or persistent immune activation caused by chronic infections such as HIV. Conversely, blocking GARP signals can amplify immune responses to antigens in the context of vaccine development or boost the immune response to infections and tumors.

## Materials and Methods

### Cell purification and culture

T_N_, T_M_, T_Nreg_ and T_reg_ cells, were purified and cultured as described before [12], [18]. Monocyte-derived dendritic cells (DCs) were generated from CD14^+^ cells from healthy donors as previously described [18]. The superantigen, staphylococcal enterotoxin B (SEB) was purchased from Sigma (St. Louis, MO). The cytokines used were from R&D Systems (Minneapolis, MN).

### Microarray gene analysis

10^6^ T cells from each subset (T_N_, T_M_, T_Nreg_ and T_reg_) were activated by plate-bound anti-CD3 antibody (OKT-3; American Type Culture Collection, Manassas, VA) and soluble anti-CD28 antibody (1 μg/ml, BD Biosciences, San Diego, CA) for 16 hours. RNA was extracted from flash frozen cells and subjected to Agilent whole Genome 4×44K arrays at a commercial service (Genus Biosystems, Northbrook, IL).

### qPCR

Total RNA was isolated and cDNA was synthesized as described before [18]. The cDNA was then used to perform qPCR using a Stratagene Mx3005P (La Jolla, CA) or Applied Biosystems 7300 apparatus (Foster City, CA). TaqMan primer and probe mixes were purchased from Applied Biosystems, and their IDs are as follows: β−actin (used as a reference gene): Hs99999903_ml; GARP: Hs00194136_m1.

### Cloning of GARP gene and construction of mutants

Full length human GARP cDNA (LRRC32) was purchased from Origene (Rockville, MD) and amplified using the primers:

5′forward: cacctctagagccatgagaccccagat;
3′reverse: cccgggcttctttaggctttata.

To construct C-terminus truncated mutant (ΔC), the following primers were used:

5′forward: cacctctagagccatgagaccccagat;
3′reverse: CCCGGGTTAGCAGGCGGCCAGCGTGGT.

The amplified DNA was then cloned into pENTR/D-TOPO® vector system (Invitrogen). The N-terminus mutant (ΔN) was cloned through PCR from WT GARP TOPO construct using the following primer sets

5′forward: gAGTactcagctgctgcggc;
3′reverse: GAGTACTGGCGGGAGCACCGAGG.

The PCR product was ligated to construct the ΔN mutant. WT GARP and the mutants were then subcloned into a HIV-derived HDV expressing RFP (Clontech Laboratories, Mountain View, CA) as marker gene as described [18]. To construct signal peptide deleted mutant (ΔSP), the following primers were used:

5′forward: GCTCTAGAGCATGGTGGACAAGAAGGTCTCGTGC;
3′reverse: CCCGGGTTAGCAGGCGGCCAGCGTGGT.

To amplify murine GARP from cDNA library, the following primers were used:

5′forward: AGCGGCCGCATTATGAGCCACCAGATCCTGCTA;
3′reverse: GGAATTCTCA GGCTTTGTATTGTTGGCTGAG.

The PCR product of murine GARP and ΔSP mutant was then subcloned into the HDV. All constructs were sequence confirmed. Lentiviruses expressing GARP or other genes used here were generated as previously described [18].

### Silencing of Foxp3 and GARP expression in primary T cells

Foxp3 or GARP shRNA-expressing lentiviral vectors with GFP as a marker was generated as previously described [12]. Two different Foxp3 shRNA sequence were tested and both showed efficient knock down of Foxp3 [12]. Both were used in experiments presented here with representatives of both (Fig. 4D) or one shRNA (sh2, Fig. 4A–C). The shRNA oligos against GARP were : sh1: GGAATTCCCTCACCTGCATCT; sh2: GGAGAACAAACTGCTCCATTT.

### FACS analysis

Cells were stained, analyzed as described before [12]. The following antibodies were used for staining: CD45RO, CD25 (both from BD Biosciences), CD62L (eBioscience, San Diego, CA). Foxp3 expression was performed with anti-Foxp3 antibody (clone 259D; Biolegend, San Diego, CA) through intracellular staining as described before [12].

### Cell proliferation, suppression and cytokine assays

Cells were labeled with CFSE (Molecular Probes, Eugene, OR) as previously described [18] and activated by DC and SEB at different concentrations. Supernatant from the culture were collected 16–40 hours later. Suppression assays were set up as previously described [12]. Cytokine production was measured using cytometric bead array (CBA; BD Biosciences) and analyzed by FlowJo (Tree Star, Ashland, OR). Data represent the mean±SEM of experiments from at least three different donors. Proliferation of the cells was assessed by monitoring expression CFSE on day 4 or day 5 post-activation.

### Detection of GARP by fluorescence microscope

WT GARP was PCR amplified with following primers:

5′forward: cacctctagagccatgagaccccagat;
3′reverse: TGGCTTTATACTGTTGG.

ΔSP mutant GARP was PCRed using primers:

5′forward: GCTCTAGAGCATGGTGGACAAGAAGGTCTCGTGC;
3′reverse: TGGCTTTATACTGTTGG.

The PCR products were ligated in pcDNA3.1/CT-GFP-TOPO® vector system (Invitrogen). Both constructs were transfected into 293T cells by lipofectamine 2000 (Invitrogen) following the manufacture's instructions. pcDNA3.1 GFP was used as control. 24 hours post transfection, cells were washed with cold PBS, fixed at 4°C in 4% paraformaldehyde for 10 min, washed one more time with PBS and mounted on glass coverslips in Aqua Poly/Mount (Polysciences, Inc, Warrigton, PA). Samples were visualized by Zeiss, Axiover 200 fluorescence microscope (Bernried, Germany), recorded by QImaging Retiga EX digital camera (Minneapolis, MN) and analyzed by Openlab 3.1.7 (Improvision, Lexington, MA). Nuclei were stained with Hoechst 33342 (Invitrogen).

### Detection of mouse GARP in murine T cell subsets

CD4^+^CD25^−^ and CD4^+^CD25^+^ T cells were purified from spleen of C57BL/6 mice by FACS. Sorted cells were stimulated with anti-CD3 and anti-CD28 antibodies (2 μg/ml each) for 24 hours. CD4^−^CD8^−^ (double negative, DN), CD4^+^CD8^+^ (double positive, DP), CD4 single positive (SP) and CD8 SP cells were purified from thymus of C57BL/6 mice by FACS. mRNA were purified and subjected to cDNA synthesis. Primers used for detection of mouse GARP are:

5′forward: TGAATTCATGAGCCACCAGATCCTGCTACTC;
3′reverse: AGCGGCCGCTCAGGCTTTGTATTGTTGGCTGAG.

Primers for detection of mouse G3PDH (glyceraldehyde-3-phosphate dehydrogenase) are described previously [28].

### Statistical Analysis

The significance was determined using One-Sample Student's t-test or One-way ANOVA.

## Supporting Information

Figure S1 Murine GARP is functionally similar to human counterpart. A) Murine GARP (mGARP) expression in mouse T cell subsets. mGARP expression in different T cell subsets and developing stages in mice as determined by semi-quantitative PCR. DN, double negative; DP, double positive; SP, single positive. G3PDH was used as loading control. One representative experiment of three is shown. (B) mGARP-transduced human TN cells displayed high levels of CD25 and CD62L expression and (C) Foxp3 expression post-activation. Cells were transduced and prepared as described in Fig. 2. M, Geometric mean. (D) Cytokine profiles of mGARP-transduced cells. Data are shown by first normalizing the control to be 100% and represent the mean±SEM of experiments from at least three different donors. Range of control production (pg/ml): IL-2, 300–1000; IFNγ, 3000–10000. *, P<0.05, n = 3; experimental versus control. (E) mGARP-transduced cells exhibited impaired proliferative capacity and (F) intermediate suppressive activity. Data are representative experiments from at least three different donors.
(0.14 MB TIF)

Figure S2 Localization of WT GARP but not ΔSP mutant to cell surface. Control GFP, WT GARP-GFP and ΔSP mutant GARP-GFP fusion protein expression in transfected 293T cells. Green, GFP; blue, Dapi. Shown are two representative merged images for WT and ΔSP mutant expressing cells.
(0.17 MB PDF)
