# Supplementary figures and images for: Identification of a Regulatory T Cell Specific Cell Surface Molecule that Mediates Suppressive Signals and Induces Foxp3 Expression
Source: PLoS One. 2008 Jul 16;3(7):e2705. doi: 10.1371/journal.pone.0002705 (PMC2442191; doi:10.1371/journal.pone.0002705)

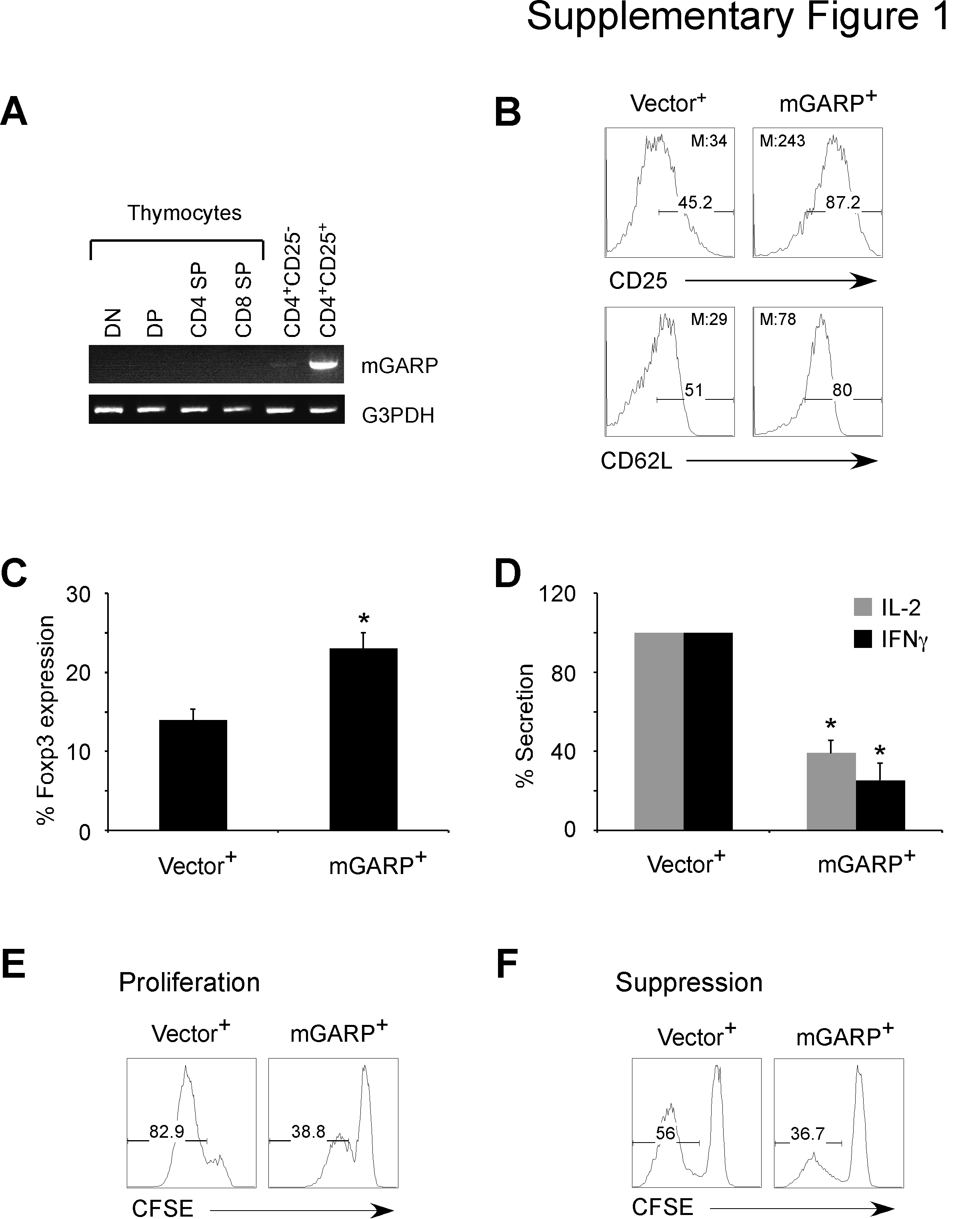

Supplement: Figure S1 — Murine GARP is functionally similar to human counterpart. A) Murine GARP (mGARP) expression in mouse T cell subsets. mGARP expression in different T cell subsets and developing stages in mice as determined by semi-quantitative PCR. DN, double negative; DP, double positive; SP, single positive. G3PDH was used as loading control. One representative experiment of three is shown. (B) mGARP-transduced human TN cells displayed high levels of CD25 and CD62L expression and (C) Foxp3 expression post-activation. Cells were transduced and prepared as described in Fig. 2. M, Geometric mean. (D) Cytokine profiles of mGARP-transduced cells. Data are shown by first normalizing the control to be 100% and represent the mean±SEM of experiments from at least three different donors. Range of control production (pg/ml): IL-2, 300–1000; IFNγ, 3000–10000. *, P<0.05, n = 3; experimental versus control. (E) mGARP-transduced cells exhibited impaired proliferative capacity and (F) intermediate suppressive activity. Data are representative experiments from at least three different donors. (0.14 MB TIF) [file pone.0002705.s001.tif]

# Supplemental Figure

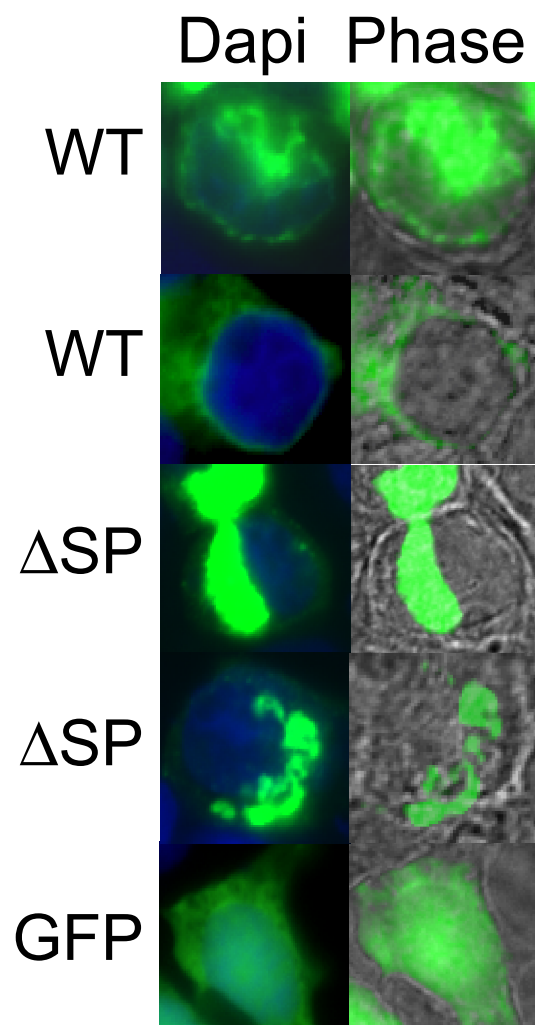

Supplement: Figure S2 — Localization of WT GARP but not ΔSP mutant to cell surface. Control GFP, WT GARP-GFP and ΔSP mutant GARP-GFP fusion protein expression in transfected 293T cells. Green, GFP; blue, Dapi. Shown are two representative merged images for WT and ΔSP mutant expressing cells. (0.17 MB PDF) [file pone.0002705.s002.pdf]
